# Marital Status and Mortality among Middle Age and Elderly Men and Women in Urban Shanghai

**DOI:** 10.1371/journal.pone.0026600

**Published:** 2011-11-02

**Authors:** Puthiery Va, Wan-Shui Yang, Sarah Nechuta, Wong-Ho Chow, Hui Cai, Gong Yang, Shan Gao, Yu-Tang Gao, Wei Zheng, Xiao-Ou Shu, Yong-Bing Xiang

**Affiliations:** 1 University of New England College of Osteopathic Medicine, Biddeford, Maine, United States of America; 2 State Key Laboratory of Oncogene and Related Genes, Shanghai Cancer Institute, Renji Hospital, Shanghai Jiaotong University School of Medicine, Shanghai, China; 3 Department of Epidemiology, Shanghai Cancer Institute, Renji Hospital, Shanghai Jiaotong University School of Medicine, Shanghai, China; 4 Division of Epidemiology, Department of Medicine, Vanderbilt Epidemiology Center, Vanderbilt-Ingram Cancer Center, Vanderbilt University School of Medicine, Nashville, Tennessee, United States of America; 5 Division of Cancer Epidemiology and Genetics, National Cancer Institute, Rockville, Maryland, United States of America; Innsbruck Medical University, Austria

## Abstract

**Background:**

Previous studies have suggested that marital status is associated with mortality, but few studies have been conducted in China where increasing aging population and divorce rates may have major impact on health and total mortality.

**Methods:**

We examined the association of marital status with mortality using data from the Shanghai Women's Health Study (1996–2009) and Shanghai Men's Health Study (2002–2009), two population-based cohort studies of 74,942 women aged 40–70 years and 61,500 men aged 40–74 years at the study enrollment. Deaths were identified by biennial home visits and record linkage with the vital statistics registry. Marital status was categorized as married, never married, divorced, widowed, and all unmarried categories combined. Cox regression models were used to derive hazard ratios (HR) and 95% confidence interval (CI).

**Results:**

Unmarried and widowed women had an increased all-cause HR = 1.11, 95% CI: 1.03, 1.21 and HR = 1.10, 95% CI: 1.02, 1.20 respectively) and cancer (HR = 1.17, 95% CI: 1.04, 1.32 and HR = 1.18, 95% CI: 1.04, 1.34 respectively) mortality. Never married women had excess all-cause mortality (HR = 1.46, 95% CI: 1.03, 2.09). Divorce was associated with elevated cardiovascular disease (CVD) mortality in women (HR = 1.47, 95% CI: 1.01, 2.13) and elevated all-cause mortality (HR = 2.45, 95% CI: 1.55, 3.86) in men. Amongst men, not being married was associated with excess all-cause (HR = 1.45, 95% CI: 1.12, 1.88) and CVD (HR = 1.65, 95% CI: 1.07, 2.54) mortality.

**Conclusions:**

Marriage is associated with decreased all cause mortality and CVD mortality, in particular, among both Chinese men and women.

## Introduction

Married individuals have been shown to have a reduced risk of mortality [1]–[3]. Two prevailing theories explaining this relationship are marriage protection and marriage selection. The marriage protection theory states that marriage provides a protective health effect through access to a network of personal social relationships, improved socioeconomic status (SES) and support, and the promotion of healthy lifestyle and behavioral choices [4]–[7]. Furthermore, bereavement and marital dissolution are associated with stress, which may increase risk of mortality [8]–[10]. In contrast, selection theory states healthier individuals are more likely to marry or stay married because of advantageous attributes like physical and psychological well being [11]. Studies have demonstrated that mortality is influenced by factors that are also associated with marital status. These factors include but are not limited to SES and behavioral factors (e.g. alcohol consumption, smoking, and diet) [5], [9], [12], [13], [14], [15], psychological distress [9], [13], [16], [17], [18], and pathophysiological mechanisms [9], [13]. For example, marriage was associated with healthier dietary behavior and improved diabetes' management because spouses are involved in meal planning, food purchases and selection, as well as preparations [5]. In contrast, women who were divorced or widowed were more likely to have decreased vegetable intake and relapse or start smoking compared to women who were married [12]. Secondly, studies have found that marriage is associated with decreased alcohol intake whereas divorce was associated with increased alcohol consumption [19], [20]. Moreover, some evidence suggests men gain more health benefits from marriage than women [2], [13], [21], [22]. For instance, a U.S. study reported that marriage provided a strong protection against increased levels of C-reactive protein in older men [23], while a British cohort study found that married men had improved midlife physical functioning [24].

Over the last three decades, China has experienced increasing divorce rates, an aging population and a reduction in family size. The divorce rate has been steadily climbing, with divorce rates of 0.4/1000 persons in 1985 to 1.85/1000 in 2009 [25]. In 2009, one in five marriages of Mainland Chinese ended in divorce, most likely driven by the high divorce rate among young Chinese [25]. Additionally, China faces a rapidly growing aging population due to improved life expectancy and large population cohorts born between 1950s and 1970s, which has resulted in an increasing population of widows [26]. Lastly, China's 1979 One Child Policy [27] has resulted in smaller families. A reduction in family size may impact elderly care. Specifically, in China, much of the elderly care is placed upon children and/or spouse. However, as the widowed and divorced population increase, much of the burden of care will be placed upon the child, without the assistance of siblings. These aforementioned population trends will influence current and future patterns in marriage, health, and mortality and highlight the need to evaluate the association of marital status and mortality among Chinese populations.

Despite numerous studies on marriage and mortality, many studies of this association have been of smaller sample size [3] and few were conducted in Asia [2], [10], [28]–[30] and specifically China [3]. Furthermore, few studies reported on specific causes of deaths, such as cancer and cardiovascular disease (CVD) in association with each marital status category. To address limitations of current literature, we used data from the Shanghai Women's Health Study (SWHS) and Shanghai Men's Health Study (SMHS) to comprehensively evaluate the association between marital status and all-cause and cause-specific mortality among middle-aged and elderly Chinese in urban Shanghai.

## Methods

### Study participants

The SWHS and SMHS are population-based prospective cohort studies in Shanghai, China. Study design and methods have been described previously [31], [32]. In short, seven study communities (for women) and eight study communities (for men) were selected on the basis of their similarity with respect to disease rates and demographic characteristics to urban Shanghai. During 1996 and 2000, all females aged 40 to 70 years who were permanent residents in the study communities were approached by community health workers or study staff to evaluate interest in the study; 74,942 women were eligible and agreed to participate (baseline response rate = 92%). Similarly, the SMHS enrolled 61,500 men aged 40–74 years from 2002 to 2006 (baseline response rate = 75%). Baseline recruitment activities, including an interview and anthropometric measurements, were conducted at participant homes by trained interviewers. Structured questionnaires were used to obtain information on demographics, diet, lifestyle habits, reproductive history (for women only), and medical history. The validity and reliability of physical activity and food frequency questionnaires have been assessed and reported in previous publications [33], [34].

### Definition of marital status

Marital status was obtained from the baseline questionnaire for women and men. The initial baseline questionnaire for the SMHS omitted marital status, but was corrected. Thus, only a subset of 53,437 men had marital status information collected at baseline and they were included in the current analysis. We defined marital status as married and unmarried (never married, separated/divorced, and widowed). We excluded 77 SWHS participants and 1,264 SMHS participants from the analysis due to missing baseline characteristics. Eight women and 26 men were lost to follow-up shortly after baseline recruitment and were excluded from analysis. Thus 74,857 women and 52,147 men were included in the current analysis.

### Outcome assessment

Follow-up included biennial in-person follow-up surveys and record linkage to the Shanghai Cancer Registry and Shanghai Municipal Registry of Vital Statistics. Outcomes data through December 31^st^, 2009 for women and December 31^st^, 2008 for men were used for the current analysis. Follow-up for mortality outcomes was >99%. Cause of death was determined using death certificates and coded according to the International Classification of Diseases, Ninth Revision (ICD-9).

### Confounding Factors and Statistical Analysis

Primary endpoints were all-cause and cause-specific deaths including deaths from CVD (ICD-9 390–459), cancer (ICD-9 140–208), and non-cancer/non-CVD causes (ICD-9 0–139, 209–389, and 460–999). Major specific CVD deaths (coronary artery disease (CAD) [ICD-9 codes 410–414 and 429] and stroke (CVA) [ICD-9 430–438]) were assessed.

Age-specific person years were calculated from participants' cumulative survival time where each observation was a record for every additional year beyond their age-specific entry at baseline date to the date of death or study end-date and then summed over the study period. Age-adjusted means and proportions for baseline characteristics were presented by marital status. Statistical testing for differences by marital status was conducted using analysis of covariance for continuous variables and Chi-square analysis for categorical variables. We compared mortality for each unmarried state (never married, separated/divorced, and widowed) and then all unmarried states combined (not married) with those who were married (reference group).

Gender-specific Cox proportional hazards regression models were used to estimate age- and multivariable-adjusted hazard ratios (HRs) with 95% confidence intervals (CIs) for all-cause and cause-specific mortality associated with marital status. Age was treated as a continuous variable in the analysis. We selected a priori baseline characteristics shown to be associated with marital status and/or mortality as potential confounding factors from the baseline survey [5], [9], [12]–[18], [35]–[39]. Thus, baseline characteristics selected as potential confounding factors included: 1) SES (educational attainment [≤elementary, middle school, high school, >high school], income [≤20,000 Chinese Yen (CNY), 20,000–29,999 CNY and ≥30,000 CNY annual income/family for women; ≤1,000 CNY, 1,000–1,999 CNY, and ≥2,000 CNY monthly income/person for men] and occupation [housewife (women only), manual, clerical, and professional]); 2) behavioral factors (BF) (total physical activity level [standard metabolic equivalents (METs) as MET-hr/day in tertiles; 1 MET-hr = 15 minutes of moderate intensity activity] [40], [41], body mass index (BMI) [<18.5 kg/m^2^;18.5–24.99 kg/m^2^; 25.0–29.99 kg/m^2^; ≥30.0 kg/m^2^] [42], waist to hip ratio (WHR) [median cutoff, low and high], smoking status [ever/never], alcohol use [ever/never], and Ginseng use [ever/never]); 3) dietary factors (total energy intake [kcal in tertiles], red meat intake [g/day in tertiles], and vegetable and fruit intake [g/day in tertiles]); 4) reproductive factors (women only) (hormone replacement therapy [yes/no] and menopausal status [pre- and post-menopausal]); and 5) baseline history of chronic disease (hypertension (HTN) [yes/no], diabetes mellitus (DM) [yes/no], baseline CVD [yes/no], and all other chronic illness [yes/no]). BMI (BMI = weight (kg)÷height (m^2^)) was a general measure of adiposity whereas WHR (WHR = waist circumference÷hip circumference) was a measure of central adiposity. Vegetable and fruit intakes were assessed separately and then combined into one variable for multivariable analyses. We then evaluated the associations of each selected baseline characteristic with both marital status and mortality. Logistic regression analysis was used to investigate the relationship between marital status and baseline characteristics and Cox regression analysis was used to analyze the relationship between mortality and baseline characteristics. Of the aforementioned potential confounding factors, menopausal status, alcohol use, Ginseng use, red meat intakes (men only) and baseline CVD were not associated with both marital status and mortality. Menopausal status (for women only), red meat intake, Ginseng use, and alcohol use remained in the full model because they were variables of interest or were shown to be potential confounders in previous literatures [35], [36], [43]. Furthermore baseline history of CVD was included in the final multivariable models to adjust for the impact of chronic disease on mortality, as well as to adjust for health selection. All other potential confounding factors described above were associated with both mortality and marital status and thus included in the final fully adjusted multivariable models.

Implementing the gender-specific Cox proportional hazards regression models, a stratified risk ratio analysis was performed to better understand possible effect modification by age. For women, multivariable-adjusted HRs were calculated to assess the association between age of marriage and all-cause and cause specific mortality. Following Dupre et al.'s definition [10], early marriage (<19 years) and late marriage (>25 years) was compared to those who married on time (19–24 years) with mortality. We evaluated multi-collinearity between covariates using logistic regression analysis. Collinearity was ruled out if the variance inflation factor (VIF) was <10, tolerance value was >0.1, and the conditional index was small. We examined the proportional hazards assumption by testing the statistical significance of time-dependent marital status variable and found no evidence for apparent departure from the assumption of proportional hazards. Statistical analyses were performed by using Statistical Analysis Software (SAS) (version 9.2; SAS Institute Inc., Cary, NC, USA). All statistical tests were based on two-sided probability and P-values ≤0.05 were considered statistically significant.

## Results

After a median follow-up of 11.21 (standard deviation (SD) 1.56) years and accumulated 822,830 person-years, the SWHS had 4,116 total deaths; 1,875 from cancer, 1,177 from CVD, and 1064 from non-cancer/non-CVD causes. CVD-specific deaths included stroke (n = 655) and CAD (n = 368). The SMHS had a median follow-up time of 4.55 (SD 1.19) years and accumulated 233,907 person-years. There were 1,575 total deaths with 692 from cancer, 517 from CVD and 366 from non-cancer/non-CVD causes. CVD-specific deaths included stroke (n = 254) and CAD (n = 194).

Table 1 shows baseline by marital status (Table 1). In our analysis, 88.8% of women and 97.3% of men were married, 0.9% of women and 1.3% of men were never married, 2.9% of women and 0.8% of men were separated/divorced, and 7.5% of women and 0.6% of men were widowed. Compared to married persons, widowed participants were about 10 years older, while never married and separated/divorced persons were 3–8 years younger. Married participants reported having higher incomes than unmarried participants. Married women were less educated than never married or separated/divorced women and half were employed as manual workers. Never married women were more educated and a higher proportion held technical/professional jobs, but were more likely to report lower family incomes than married women. In contrast, married men were more educated with a greater proportion reporting professional/technical occupations compared to unmarried men.

**Table 1:** Age adjusted characteristics at baseline by marital status, the Shanghai Men's Health Study, 2002–2008 (n = 52,147), and Shanghai Women's Health Study, 1996–2009 (n = 74,857).

			Women					Men		
Marital status	Married	Never Married	Sep/Div	Widowed	p value‡	Married	Never Married	Sep/Div	Widowed	p value‡
	n = 66,436	n = 649	n = 2,181	n = 5,591		n = 50,722	n = 701	n = 404	n = 320	
Age, mean (SD)	51.79 (0.03)	49.16 (0.34)	49.61 (0.18)	62.03 (0.11)	<0.0001	55.77 (0.04)	47.79 (0.37)	51.28 (0.50)	65.16 (0.55)	<0.0001
***Socioeconomic factors***										
Educational level achieved (%)										
≤Elementary school	20.88	14.18	19.18	25.69		6.61	8.44	6.18	11.03	
Middle school	37.27	27.42	35.55	38.00		33.48	45.33	38.23	33.62	
High school	28.14	30.28	28.65	26.28		35.60	29.29	36.34	40.30	
≥Prof/Tech/College	13.71	28.11	16.62	10.02	<0.0001	24.31	16.94	19.25	15.05	<0.0001
Ever employment (%)										
Manual workers	50.20	40.79	51.12	51.36		50.98	63.42	61.20	54.18	
Prof/Tech	29.01	41.72	28.79	25.00		27.14	15.97	20.38	19.54	
Clerical	20.45	16.20	19.83	23.12		21.88	20.61	18.41	26.28	
House Wife	0.33	1.29	0.26	0.51	<0.0001	-----	-----	-----	-----	<0.0001
Income (%)^a^										
Low	52.84	72.03	72.20	75.55		54.21	71.60	62.76	59.65	
Middle	28.93	19.19	17.04	15.82		35.74	26.96	30.31	33.76	
High	18.23	8.78	10.76	8.63	<0.0001	10.05	1.44	6.93	6.59	<0.0001
***Reproductive factors***										
Menopause (%)	49.52	50.62	49.80	51.01	0.0455	-----	-----	-----	-----	-----
HRT (%)	2.16	1.59	2.87	1.37	0.0002	-----	-----	-----	-----	-----
***Behavioral factors***										
Ever smoking (%)	2.59	0.46	6.45	3.68	<0.0001	69.26	70.26	73.64	77.22	0.0002
Pack Years:										
0.1-15.9 Pack Years	-----	-----	-----	-----	-----	23.82	20.23	18.45	30.00	
16.0-31.4 Pack Years	-----	-----	-----	-----	-----	27.41	23.42	30.61	25.85	
≥31.5 Pack Years	-----	-----	-----	-----	-----	18.01	26.61	24.58	21.38	0.0002
Ever alcohol intake (%)	2.19	1.38	3.55	2.33	<0.0001	33.30	27.37	38.64	37.14	0.0067
Alcohol drinks/day										
0.1-1.0	-----	-----	-----	-----	-----	6.72	5.37	5.06	4.94	
1.1-2.0	-----	-----	-----	-----	-----	13.48	9.11	15.38	16.73	
≥2.0	-----	-----	-----	-----	-----	13.10	12.89	18.21	15.46	0.0002
Ever Ginseng intake (%)	29.42	21.56	29.77	32.85	<0.0001	33.06	17.14	26.19	31.48	<0.0001
BMI (kg/m^2^), mean (SE)	24.03 (0.01)	23.46 (0.13)	23.61 (0.07)	24.02 (0.05)	<0.0001	23.76 (0.01)	23.09 (0.12)	23.02 (0.15)	23.42 (0.17)	<0.0001
WHR, mean (SE)	0.8112 (0.0002)	0.8070 (0.0020)	0.8041 (0.0011)	0.8116 (0.0007)	<0.0001	0.9006 (0.0003)	0.8958 (0.0022)	0.8942 (0.0028)	0.8970 (0.0032)	<0.0001
MET-h/day, mean (SE)	106.83 (0.18)	87.49 (1.77)	102.00 (0.97)	105.79 (0.63)	<0.0001	59.66 (0.15)	63.99 (1.26)	65.75(1.65)	67.40 (1.86)	<0.0001
***Dietary factors, mean (SE)***										
Total energy intake (kcal/day)	1,675.56 (1.57)	1,710.40 (15.88)	1,722.66 (8.67)	1,661.50 (5.65)	<0.0001	1909.38 (2.12)	1858.21 (18.11)	1915.13 (23.77)	1891.90 (26.76)	0.0389
Vegetable intake (g/day)	294.94 (0.66)	338. 10 (6.68)	339.72 (3.65)	295.77 (2.38)	<0.0001	344.85 (0.85)	309.52 (7.26)	338.38 (9.53)	316.50 (10.73)	<0.0001
Fruit intake (g/day)	266.57 (0.69)	244.72 (6.92)	269.02 (3.78)	242.41 (2.46)	<0.0001	153.28 (0.55)	116.21 (4.72)	122.68 (6.20)	119.48 (6.98)	<0.0001
Red Meat intake (g/day),	50.76 (0.14)	51.73 (1.42)	57.16 (0.78)	50.58 (0.51)	<0.0001	62.95 (0.19)	58.51 (1.64)	68.90 (2.16)	68.48 (2.43)	0.0002
***History of chronic disease***										
HTN (%)	23.93	18.77	18.82	24.18	<0.0001	30.56	32.20	28.20	26.61	0.1447
DM (%)	4.62	4.60	3.88	5.20	0.2079	6.47	4.55	8.57	5.44	0.8274
Baseline CVD (%)	15.72	17.04	13.53	15.70	0.0391	16.54	16.21	18.99	18.98	0.3588
Other chronic disease (%)	41.38	42.33	45.06	37.98	<0.0001	37.45	32.69	33.59	35.23	0.0113

Abbreviations: SD (Standard Deviation), SE (Standard Error), HRT (hormone replacement therapy), BMI (body mass index), WHR (waist to hip ratio), MET (metabolic equivalents), HTN (hypertension), DM (diabetes mellitus), CHD (coronary heart disease), CNY (Chinese Yuan).

a Income –Men (Monthly income/person Low <1,000 CNY, Middle 1,000–1,999 CNY, High ≥2,000 CNY); Women (Annual income/Family Low <20,000 CNY, Middle 20,000–29,999 CNY, High >30,000 CNY).

‡ Statistical significance was tested using chi square test for categorical variables and ANOVA for continuous variables.

Overall, less than 7% of the women reported ever smoking and less than 4% of women reported having ever consumed alcohol. In contrast, about 69–77% of men reported tobacco smoking and about 1/3 of men reported alcohol use. Furthermore, separated/divorced and widowed persons reported the highest proportion of tobacco smoking and alcohol use. Divorced participants had higher total energy and red meat intakes compared to married participants. Divorced women reported higher vegetable and fruit intakes compared to married women, while married men reported the highest consumption of vegetables and fruits compared to unmarried men. Married women were the most physically active, while married men were the least physically active. We found statistically significant effect modification by sex (p<0.05) for the association of marital status and mortality; hence, results are presented separately for women and men.

Table 2 shows multivariable-adjusted HRs for the association between marital status and all-cause and selected cause-specific mortality for women and men. Among women, not being married was associated with excess all-cause mortality (HR = 1.12, 95% CI: 1.03, 1.21) and cancer mortality (HR = 1.17, 95% CI: 1.04, 1.32). Compared to being married, widowhood was associated with elevated all-cause (HR = 1.10, 95% CI: 1.01, 1.20) and cancer (HR = 1.18, 95% CI: 1.04, 1.34) mortality, being separated/divorced was associated with elevated CVD mortality (HR = 1.47, 95% CI: 1.01, 2.13), and never being married was associated with elevated all-cause mortality (HR = 1.46, 95% CI: 1.03, 2.09). As shown in Table 2, among men, not being married was associated with increased all-cause mortality (HR = 1.45, 95% CI: 1.12, 1.88) and CVD mortality (HR = 1.65, 95% CI: 1.07, 2.54). Being divorced/separated was associated with the greatest magnitude of excess death from all-causes (HR = 2.45, 95% CI: 1.55, 3.86), CAD (HR = 4.01, 95% CI: 1.27, 12.67) and non-cancer/non-CVD causes (HR = 3.79, 95% CI: 1.78, 8.07). Never being married was associated with a 2.6-fold increased risk of CVD mortality (HR = 2.56, 95% CI: 1.20, 5.46); but was not significantly associated with other mortality outcomes. No significant associations were seen between martial status and cancer mortality in men. Furthermore, being widowed was not significantly associated with all-cause or cause-specific mortality.

**Table 2:** Hazard Ratio (HR) for the association of marital status and all-cause mortality and selected cause-specific mortality, the Shanghai Men's Health 2002–2008 (n = 52,147) and Shanghai Women's Health Study 1996–2009 (n = 74,857).

			Women					Men		
	Married	Never Married	Sep/Div	Widowed	Not Married	Married	Never Married	Sep/Div	Widowed	Not Married
	n = 66,436	n = 649	n = 2,181	n = 5,591	n = 8,421	n = 50,722	n = 701	n = 404	n = 320	n = 1,425
**All causes**										
No. of deaths	3,233	31	92	760	883	1,515	15	19	26	60
Multivariate HR (95% CI)‡	1.00	1.46(1.03,2.09)	1.14(0.92, 1.40)	1.10(1.02,1.20)	1.12(1.03,1.21)	1.00	1.56(0.93,2.60)	2.45(1.55,3.86)	1.09(0.74,1.61)	1.45(1.12,1.88)
**All cancers**										
No. of deaths	1,505	16	42	312	370	669	6	7	10	23
Multivariate HR (95% CI)‡	1.00	1.52(0.93,2.49)	1.05(0.77,1.43)	1.18(1.04,1.34)	1.17(1.04,1.32)	1.00	1.33(0.59,2.98)	1.97(0.93,4.16)	1.00(0.54,1.88)	1.28(0.84,1.94)
**All CVD**										
No. of deaths	898	6	29	244	279	495	7	5	10	22
Multivariate HR (95% CI)‡	1.00	1.27(0.57,2.84)	1.47(1.01,2.13)	1.05(0.91,1.21)	1.09(0.95,1.25)	1.00	2.56(1.20,5.46)	2.12(0.87,5.13)	1.21(0.65,2.28)	1.65(1.07,2.54)
Stroke										
No. of deaths	512	3	16	124	143	245	4	1	4	9
Multivariate HR (95% CI)‡	1.00	1.10(0.35,3.43)	1.44(0.88,2.38)	0.93(0.76,1.14)	0.97(0.81,1.18)	1.00	2.41(0.89,6.59)	0.76(0.11,5.44)	1.03(0.38,2.78)	1.31(0.67,2.56)
CAD										
No. of deaths	274	1	9	84	94	185	0	3	6	9
Multivariate HR (95% CI)‡	1.00	0.75(0.11,5.36)	1.55(0.80,3.03)	1.16(0.90,1.50)	1.19(0.93,1.51)	1.00	0.00 (-----)	4.01(1.27,12.67)	1.87(0.82,4.27)	1.89(0.96,3.71)
**Non-cancer/Non-CVD**										
No. of deaths	830	9	21	204	234	351	2	7	6	15
Multivariate HR (95% CI)‡	1.00	1.60(0.83,3.09)	0.99(0.64,1.53)	1.06(0.90,1.24)	1.07(0.92,1.24)	1.00	0.86(0.21,3.50)	3.79(1.78,8.07)	1.06(0.47,2.38)	1.52(0.91,2.57)

Abbreviations: BMI (body mass index), CI (confidence interval), CVD (cardiovascular disease), DM (diabetes mellitus), HRT (hormone replacement therapy), HTN (hypertension), HR (Hazard ratio), SES (socioeconomic status), WHR (waist to hip ratio).

‡ Adjusted for: age, SES (income, education, occupation), behavioral factors (BMI, WHR, physical activity, ever smoke, ever alcohol drinking, ever Ginseng use), reproductive factors (HRT, menopause, *women only*), dietary factors (total energy intake, vegetable and fruit intake, and red meat intake), chronic disease at baseline (HTN, DM, baseline CVD, and all other chronic disease).

Adjustments of all the previously mentioned risk factor groups (SES factors, behavioral factors, dietary factors, reproductive factors, and baseline chronic disease history) explained only 43% (women) and 38% (men) of the elevated mortality risk associated with marital status and all-cause mortality. Of all the risk factor groups, SES explained the greatest proportion of elevated risks associated with all-cause (33%) and CVD (41%) mortality for women. For men, SES contributed to a smaller proportion of risk reduction for all-cause (28%) and CVD mortality (24%).

Table 3 shows the association between marital status and mortality stratified by age. In women age ≥65 years, not being married was associated with significantly increased risk of all-cause (HR = 1.11, 95% CI: 1.02, 1.21) and cancer (HR = 1.19, 95% CI: 1.04, 1.36) mortality. These associations were in the same direction, albeit not statistically significant among younger women. Additionally, being divorced/separated was significantly associated with increased all-cause and CVD mortality among women ≥65 years of age, but not among women <65 years. In younger women, widowhood was associated with a greater magnitude of increased all-cause and cancer mortality compared to older women.

**Table 3:** HRs for the association of marital status and all-cause mortality by age, the Shanghai Men's Health Study 2002–2008 (n = 52,147), and Shanghai Women's Health Study 1996–2009 (n = 74,857).

			Women					Men		
	Married	Never Married	Sep/Div	Widowed	Not Married	Married	Never Married	Sep/Div	Widowed	Not Married
	732,712 PY	7,064 PY	23,789 PY	59,265 PY	90,118 PY	227,829 PY	3,027 PY	1,684 PY	1,367 PY	6,078 PY
**Ages 40–64 yrs**										
*Person-years*	554,771	6,287	20,081	17,380	43,748	164,381	2,846	1,513	398	4,757
All-cause										
No. of deaths	1,115	21	34	75	130	460	13	9	2	24
Multivariate HR (95% CI)‡	1.00	1.53(0.99,2.37)	0.92(0.65,1.30)	1.25(0.98,1.58)	1.17 (0.97–1.41)	1.00	1.82(1.04,3.17)	1.93(0.99,3.74)	1.71(0.43,6.86)	1.85(1.22,2.80)
All cancers										
No. of deaths	658	10	20	45	75	232	5	1	0	6
Multivariate HR (95% CI)‡	1.00	1.28(0.68,2.41)	0.93(0.59,1.45)	1.38(1.01,1.88)	1.21(0.95,1.54)	1.00	1.51(0.62,3.71)	0.44(0.06,3.16)	0.00 (-----)	0.96(0.43,2.18)
CVD										
No. of deaths	196	2	5	15	22	120	6	4	0	10
Multivariate HR (95% CI)‡	1.00	0.83(0.20, 3.38)	0.80(0.33,1.94)	1.10(0.64,1.89)	0.98(0.63,1.54)	1.00	3.39(1.46,7.82)	3.24(1.19,8.84)	0.00 (-----)	3.03(1.57,5.85)
Non-Cancer/CVD										
No. of deaths	261	9	9	15	33	108	2	4	2	8
Multivariate HR (95% CI)‡	1.00	2.54(1.29,5.01)	1.02(0.52,1.99)	1.05(0.62,1.79)	1.24(0.86,1.79)	1.00	0.94 (0.23,3.85)	3.41(1.25,9.35)	8.23(2.02,33.58)	2.29(1.10,4.76)
**Ages 65+yrs**										
*Person-years*	177,941	777	3,708	41,885	46,370	63,448	181	171	969	1,321
All-cause										
No. of deaths	2,118	10	58	685	753	1,055	2	10	24	36
Multivariate HR (95% CI)‡	1.00	1.21(0.65,2.25)	1.33(1.02,1.73)	1.09(1.00,1.19)	1.11(1.02,1.21)	1.00	0.75(0.19,3.01)	3.22(1.72,6.02)	1.04(0.69,1.56)	1.24(0.89,1.74)
All cancers										
No. of deaths	847	6	22	267	295	437	1	6	10	17
Multivariate HR (95% CI)‡	1.00	1.83(0.82,4.11)	1.24(0.81,1.89)	1.17(1.02,1.35)	1.19(1.04,1.36)	1.00	0.78(0.11,5.56)	4.69(2.08,10.60)	1.09(0.58,2.05)	1.45(0.89,2.36)
CVD										
No. of deaths	702	4	24	229	257	375	1	1	10	12
Multivariate HR (95% CI)‡	1.00	1.52(0.57,4.07)	1.76(1.17,2.64)	1.05(0.90,1.22)	1.10(0.95,1.27)	1.00	1.14(0.16,8.18)	0.86(0.12,6.16)	1.23(0.66,2.32)	1.18(0.66,2.11)
Non-Cancer/CVD										
No. of deaths	569	0	12	189	201	243	0	3	4	7
Multivariate HR (95% CI)‡	1.00	0.00 (-----)	0.98(0.55,1.73)	1.04(0.88,1.23)	1.02(0.87,1.20)	1.00	0.00 (-----)	4.10(1.30,12.98)	0.69(0.25,1.85)	1.00(0.47,2.14)

Abbreviations: BMI (body mass index), CI (confidence interval), CVD (cardiovascular disease), DM (diabetes mellitus), HRT (hormone replacement therapy), HTN (hypertension), HR (Hazard ratio), SES (socioeconomic status), WHR (waist to hip ratio), PY (person years).

‡ Adjusted for: age, SES (income, education, occupation), behavioral factors (BMI, WHR, physical activity, ever smoke, ever alcohol drinking, ever Ginseng use), reproductive factors (HRT, menopause, *women only*), dietary factors (total energy intake, vegetable and fruit intake, and red meat intake), chronic disease at baseline (HTN, DM, baseline CVD, and all other chronic disease).

Unlike women, men experienced a greater magnitude of association between not being married and all-cause (HR = 1.85, 95% CI: 1.22, 2.80), CVD (HR = 3.03, 95% CI: 1.57, 5.85), and non-cancer/non-CVD (HR = 2.29, 95% CI: 1.10, 4.76) mortality at younger ages (40–64 years). Being never married was associated with increased all-cause mortality and CVD mortality among younger, but not older men. Regardless of age group, divorce was associated with elevated all-cause and non-cancer/non-CVD mortality in men. In younger men, divorce was associated with a 3.2 fold elevated CVD mortality. However, in men 65 years of age and older, divorce was associated with a significant 4.7 fold increased risk of cancer mortality (Table 3).

Table 4 shows multivariable-adjusted HRs for the association between age of marriage and all-cause and cause-specific mortality. The analysis confined to women only. Marrying early (<19 years) was associated with a 13%–22% increased risk of mortality, whereas marrying late (>25 years) was associated with a reduced risk of all-cause mortality after adjustments for potential confounding factors, excluding SES factors. However, these significant associations disappeared after adjustments for SES.

**Table 4:** HRs between marriage age and all-cause mortality and cause-specific mortality, Shanghai Women's Health Study 1996–2009 (n = 74,208*).

		Age at first marriage	
	Ages 19–25	Early, age ≤18	Late, age >25
Causes of deaths	(n = 31,481)	(n = 3,910)	(n = 38,817)
All causes			
No. of deaths	2,390	584	1,111
Model 1 HR (95% CI)^a^	1.00	1.21 (1.11–1.33)	0.90 (0.83,0.97)
Model 2 HR (95% CI)^b^	1.00	1.12 (1.02,1.23)	0.91 (0.84,0.99)
Model 3 HR (95% CI)^c^	1.00	1.04 (0.95–1.14)	1.01 (0.93,1.09)
All cancer			
No. of deaths	1,045	222	592
Model 1 HR (95% CI)^a^	1.00	1.14 (0.98,1.32)	0.91 (0.81,1.01)
Model 2 HR (95% CI)^b^	1.00	1.09 (0.94,1.27)	0.93 (0.83,1.04)
Model 3 HR (95% CI)^c^	1.00	1.05 (0.90,1.22)	0.97 (0.86,1.09)
CVD			
No. of deaths	740	198	233
Model 1 HR (95% CI)^a^	1.00	1.22 (1.04,1.42)	0.78 (0.66,0.91)
Model 2 HR (95% CI)^b^	1.00	1.09 (0.93,1.28)	0.81 (0.69,0.95)
Model 3 HR (95% CI)^c^	1.00	1.00 (0.85,1.17)	0.93 (0.79,1.09)
Non-cancer/CVD			
No. of deaths	605	164	286
Model 1 HR (95% CI)^a^	1.00	1.30 (1.09,1.55)	0.99 (0.85,1.16)
Model 2 HR (95% CI)^b^	1.00	1.20 (1.00,1.43)	0.99 (0.85,1.15)
Model 3 HR (95% CI)^c^	1.00	1.08 (0.90,1.28)	1.15 (0.98,1.35)

Abbreviations: BMI (body mass index), CI (confidence interval), CVD (cardiovascular disease), DM (diabetes mellitus), HRT (hormone replacement therapy), HTN (hypertension), HR (Hazard ratio), SES (socioeconomic status), WHR (waist to hip ratio).

*Sample size is reduced to include only women who were ever married (married, separated/divorced, widowed).

a Model 1: Adjusted for age only.

b Model 2: Multivariate model ***without*** SES(income, education, occupation). Adjustments include: age, behavioral factors (BMI, WHR, MET, ever smoke, ever alcohol drinking, ever Ginseng use), reproductive factors (HRT, menopause), dietary factors (total energy intake, vegetable & fruit intake, and red meat intake), chronic disease at baseline (HTN, DM, baseline CVD, and all other chronic disease).

c Model 3: Multivariate model ***with*** SES. Adjustments include: age, SES, behavioral factors, dietary factors, reproductive factors, and history of chronic disease.

## Discussion

To our knowledge, this is the first comprehensive study to evaluate the association between marital status and mortality in China. In this large prospective study, we found that not be married was associated with increased risk of mortality among both women and men. The association appears stronger in men than in women. Further, we found that associations were modified by age. For example, unmarried women had a greater magnitude of increased mortality from all-causes and cancer at older ages compared to younger ages. In contrast, unmarried men experienced greater risk of all-cause, CVD and non-cancer/non-CVD mortality at younger ages as compared to older ages.

Of all the factors considered to explain the association between marital status and mortality, SES explained the greatest proportion of risk reduction in men and women for all-cause mortality. These findings support the argument that marriage may protect health by additional mechanisms, such as providing financial stability [10]. However, in our study, the marriage and mortality association remained after adjustment for SES, behavioral factors, diet, and baseline chronic disease history. Additionally, spouses promote positive health behaviors, such as encouraging screening exams and regular doctor visits thereby influencing morbidity and mortality [4], [21], [41], [44]. Marriage's protective influence on all-cause, cancer, and CVD mortality may be due to high quality social support associated with better immune responses and lower stress indicators [9], [21], [45].

Consistent with previous studies [28], [29], we found that the relationship between marital status and mortality was significantly modified by sex. Specifically, we found that Chinese men appeared to benefit more from marriage protection than women. A meta-analysis conducted by Manzoli, L. et al., 2007 concluded that marriage was not gender dependent [3]; however, subsequent studies have reported some evidence for a gender difference [28], [29]. The gender differential may arise from differences in social control of behavior [5], [12], health behaviors, exposures to stress, and use and availability of support networks [46]–[48]. Men typically rely on the wife as the main support source, whereas women have several close confidants [46], [47]. Other considerations are differences in genetics and hormone factors.

Our findings indicate that not being married is associated with increased mortality, in particular for older women (≥65 years of age). Furthermore, amongst women and not men, widowhood was associated with elevated risk of death from all causes and cancer. According to the 2010 Census released by the National Bureau of Statistics, the proportion of Chinese people aged ≥60 years old grew to 13.26% of the 1.34 billion people residing in China, up 2.93% from the 2000 census [49]. China's aging population is expected to increase to about 330 million by the year 2050, creating a large number of people living without a spouse [50]. Compared to men, women have longer life expectancies and may marry at younger ages; hence, women are more likely to be widowed [51]. In 1990, among Chinese people ages 65–79 years, 37.4% of men and 66.5% of women were widowers [26]. Many elderly women in our cohort lived during periods with a predominantly patriarchal, patrilineal, and patrilocal society [52]. Thus, SES for these women may be more tied to marriage than women who marry today. Disproportionate excess mortality for women who are widowed may reflect differences in lifestyle, access to medical care, and social support after retirement and loss of a spouse. Furthermore, previous studies indicate that older widowed women and men are less affected by bereavement than younger ones [1], [53]. Furthermore, although not addressed in our study, the risk of dying is greatest within six months from the death of a spouse [54].

Shanghai has the 6^th^ highest divorce rate in China [25], due to increased financial independence and improved SES among women, a more liberal view of divorce, less forceful pressure from senior relatives and changes in marriage laws that make obtaining a divorce easier [55], [56]. In our study, divorced men generally experienced a larger magnitude of excess of all-cause and non-cancer/non-CVD mortality than other unmarried states when compared to married men. The relationship between divorce and CVD mortality was also significant in women. These findings have been seen consistently for men in previous studies, but are less consistent for women [2], [13], [57], [58]. Health behavior, psychological and marital distress, metabolic dysregulation, emotional stress and depression accompanying marital transitions may explain the relationship between all-cause and CVD mortality and divorce [13], [59]–[62].

Though based on a sample size, we found significant increased CVD mortality among never married men and increased all-cause mortality among never married women. Our results suggest that marriage reduces mortality for both men and women, where marital dissolution such as divorce and widowed hood are both associated with elevated mortality risks. Life-course theory suggests the impact of marital dissolution and marriage protection on survival is anchored in young adulthood [10], [63]. Earlier marriages are marked by challenges of early parental responsibilities, psychological distress, maladaptive behaviors, and economic hardships [10], [63], [64], which may disrupt normal developmental trajectories and subsequently impact mortality. In our analysis (among women only), we found that marrying early (<19 years) was associated with increased risk of mortality, whereas marrying late (>25 years) was associated with reduced all-cause mortality; however, consistent with life-course theory, the significant relationship disappeared after SES adjustments in our multivariable analysis [65].

The study has several limitations. First, marital status was measured only once; hence, we cannot account for marital history and transitions. Second, for the men's cohort, marital status was not obtained until corrections were made to the baseline questionnaire; thus, 8,063 men missing data on marital status were excluded from our study. Third, due to the age range of our cohort, about 1/3^rd^ of our cohort has chronic disease at baseline. As a result, it was not feasible to exclude cases with pre-existing chronic disease without impacting the number of cases included for analysis. Thus, although we adjusted for history of chronic disease at baseline in our model, health selection cannot be excluded. Furthermore, although we adjusted for a multitude of confounding factors, the influence of unmeasured potential confounding factors can not be measured. Fourth, we were unable to consider depression and marital quality in our study, which may influence estimates of the effect of marriage on mortality. Lastly, it is important to interpret the results of the SMHS with some caution, in particular for the results stratified by age and for cause-specific deaths, due to small sample sizes.

The strengths of our study include the population-based prospective design and large sample size. Additionally, the study is one of few studies focusing on a population in a non-western country, Mainland China. Due to the large sample size, we were able to clarify the associations between marital status and mortality from all causes, cancer, CVD and non-cancer/non-CVD causes.

Our study results indicate a great mortality disparity between those who are unmarried and married in middle-aged and elderly women and men in Shanghai. The mortality disparity amongst unmarried elderly persons poses public health challenges for now and in the future. In China, reductions in family size (3.44 persons in 2000 vs. 3.10 persons per family in 2010) [49] may undermine traditional support mechanisms for elderly Chinese, where the care of the elderly falls upon young adults without assistance from siblings. Our results support the need for access to long-term care facilities, medical care, and financial and social support for elderly unmarried persons. Additionally, access to bereavement counseling and support should be provided, even if the loss of a spouse occurs at younger ages. Furthermore, public health policies should promote a healthy lifestyle, including participation in physical activity, healthy dietary choices, and smoking cessation in unmarried populations with special attention given to young never married persons, widowed women, and separated/divorced men. Future studies should examine how marital transitions and quality impact mortality risk.
